# Asia’s dementia burden: from epidemiological description to prevention-oriented neurology

**DOI:** 10.1016/j.tjpad.2026.100627

**Published:** 2026-06-20

**Authors:** Haoyang Hu

**Affiliations:** a Department of Neurology, The First Medical Center, Chinese PLA General Hospital, Beijing, China; b Medical School of Chinese PLA, Beijing 100853 China

Alzheimer’s disease and other dementias (ADODs) are becoming one of the most important neurological and public health challenges in ageing societies. The recent study by Zhang and colleagues provides a timely comparative assessment of ADODs burden in Japan, China, and India from 1990 to 2023, using estimates from the Global Burden of Disease Study 2023 [1]. By comparing three countries at different demographic stages, the study offers useful evidence for understanding how dementia burden evolves under different patterns of ageing, population growth, epidemiological transition, and risk-factor exposure.

A central contribution of this study is that it moves beyond a simple description of case numbers. The authors evaluated incidence, prevalence, mortality, disability-adjusted life years (DALYs), decomposition drivers, attributable risk factors, and future projections [1]. This multidimensional approach is important because dementia burden is not determined by ageing alone. It is also shaped by diagnostic capacity, vascular and metabolic health, environmental exposure, long-term care systems, and social preparedness. The projected global increase in dementia prevalence further highlights the need for countries to act before health systems become overwhelmed [3].

The contrast among Japan, China, and India is especially informative. China had the highest absolute burden and the highest age-standardized incidence, prevalence, and DALY rates among the three countries in 2023 [1]. This finding suggests that China’s dementia challenge reflects both rapid population ageing and increasing age-specific disease burden. The decomposition analysis also showed that population ageing and epidemiological change jointly contributed to the increase in DALYs in China [1]. Therefore, dementia prevention in China should not be limited to expanding geriatric services. It should also strengthen midlife prevention, early cognitive assessment, vascular-metabolic risk control, and community-based dementia management. This is consistent with previous evidence showing a substantial burden of dementia and mild cognitive impairment among older adults in China [4].

Japan offers a different perspective. As a super-aged society, Japan has long faced the consequences of population ageing, but its relatively mature health and long-term care systems may partly buffer the impact of dementia on patients and families [5]. In the study by Zhang et al., Japan showed the highest age-standardized mortality rate, and future DALYs were projected to increase substantially [1]. These findings indicate that even countries with established care systems cannot rely solely on diagnosis and institutional care. For Japan, the next priority is to maintain function and dignity in the oldest-old population, reduce caregiver burden, and shift more resources toward early prevention and integrated community support.

India represents another stage of the dementia transition. Although India had lower age-standardized rates than China and Japan, it showed the most rapid increases in mortality and DALYs [1]. This pattern may reflect population growth, improving case detection, previous underdiagnosis, limited long-term care capacity, and uneven access to medical services. The finding that household air pollution from solid fuels contributed substantially to ADODs-related DALYs in India is particularly important [1]. It reminds us that dementia prevention is not only a neurological issue but also a matter of environmental health, social equity, and primary care capacity.

The risk-factor results of this study have direct implications for prevention-oriented neurology. Ambient particulate matter pollution was the leading attributable risk factor across the three countries, while high fasting plasma glucose also made an important contribution [1]. These findings are in line with current evidence that dementia risk is influenced by multiple modifiable factors, including cardiometabolic disorders, air pollution, smoking, physical inactivity, hearing loss, depression, and limited education [2]. Neurologists should therefore participate not only in diagnosis and symptomatic treatment, but also in broader prevention strategies that integrate brain health with cardiovascular, metabolic, environmental, and social interventions.

However, the findings should be interpreted with methodological caution. First, GBD-based estimates depend on available data sources, diagnostic definitions, coding practices, and statistical modeling assumptions [1]. Cross-country comparisons may be affected by differences in diagnostic awareness and health information systems. Second, ADODs as a combined category includes Alzheimer’s disease, vascular dementia, mixed dementia, and other dementing disorders. This limits the ability to develop subtype-specific strategies. Third, projections based on historical trends may not fully capture future changes, such as blood-based biomarkers, disease-modifying therapies, air-quality policies, or major reforms in long-term care [1].

Despite these limitations, the study provides a clear message: dementia policy in Asia must become earlier, broader, and more tailored. Earlier means shifting prevention into midlife and even earlier stages of the life course. Broader means integrating neurology with primary care, chronic disease prevention, environmental policy, and social care. More tailored means that each country should respond to its own drivers of burden. China should prioritize vascular-metabolic control, air-pollution reduction, and scalable community dementia services. Japan should focus on healthy ageing, long-term care sustainability, and support for caregivers. India should strengthen awareness, early diagnosis, clean household energy, and basic dementia care capacity.

Overall, Zhang and colleagues have provided an important epidemiological foundation for dementia prevention and policy planning in Asia [1]. The next task is to translate these burden estimates into practical interventions. In the coming decades, progress in dementia control will depend not only on new drugs and biomarkers, but also on whether societies can reduce modifiable risks, detect cognitive decline earlier, and build care systems that protect patients’ dignity throughout the disease course.

## Ethics approval and consent to participate

Not applicable.

## Consent for publication

Not applicable.

## Data availability

Data will be made available on request.

## Funding

This research received no specific grant from any funding agency in the public, commercial, or not-for-profit sectors.

## Declaration of the use of generative AI and AI-assisted technologies in scientific writing and in figures, images and artwork

The author declares that no generative AI or AI-assisted technologies were used in the preparation of the manuscript text, figures, images, or artwork.

## CRediT authorship contribution statement

**Haoyang Hu:** Conceptualization, Validation, Writing – review & editing.

## Declaration of competing interest

The authors declare that they have no known competing financial interests or personal relationships that could have appeared to influence the work reported in this paper.

## References

[1] Zhang C., Chen X., Sun Y. (2026). The growing burden of dementia in Asia: comparative insights from Japan, China, and India. J Prev Alzheimer’s Dis.

[2] Livingston G., Huntley J., Liu K.Y., Costafreda S.G., Selbæk G., Alladi S. (2024). Dementia prevention, intervention, and care: 2024 report of the Lancet standing Commission. Lancet.

[3] GBD 2019 Dementia Forecasting Collaborators (2022). Estimation of the global prevalence of dementia in 2019 and forecasted prevalence in 2050: an analysis for the global burden of disease study 2019. Lancet Public Health.

[4] Jia L., Du Y., Chu L., Zhang Z., Li F., Lyu D. (2020). Prevalence, risk factors, and management of dementia and mild cognitive impairment in adults aged 60 years or older in China: a cross-sectional study. Lancet Public Health.

[5] Baba H., Nakata Y., Urakami K., Suzuki Y., Toba K. (2024). Exploring the contribution of Japan’s experience in addressing rapid aging in Asia: focus on dementia care. Glob Health Med.

